# Exosomal miR-224-5p from Colorectal Cancer Cells Promotes Malignant Transformation of Human Normal Colon Epithelial Cells by Promoting Cell Proliferation through Downregulation of CMTM4

**DOI:** 10.1155/2022/5983629

**Published:** 2022-06-30

**Authors:** Feng Wu, Jiani Yang, Guoyin Shang, Zhijia Zhang, Sijia Niu, Yang Liu, Hongru Liu, Jing Jing, Yu Fang

**Affiliations:** ^1^Department of Gastroenterology, The First Affiliated Hospital of Harbin Medical University, Harbin, Heilongjiang 150001, China; ^2^Department of Gastrointestinal Medical Oncology, Harbin Medical University Cancer Hospital, Harbin, Heilongjiang 150081, China; ^3^Pharmacy Intravenous Admixture Services, The 2nd Affiliated Hospital of Harbin Medical University, Harbin, Heilongjiang 150001, China; ^4^Department of Medical Oncology, Harbin Medical University Cancer Hospital, Harbin Medical University, Harbin, Heilongjiang 150000, China; ^5^Department of Phase I Clinical Trial Ward, Harbin Medical University Cancer Hospital, Harbin Medical University, Harbin, Heilongjiang 150081, China

## Abstract

**Background:**

Interactions between malignant cells and neighboring normal cells are important for carcinogenesis. In addition, cancer cell-derived exosomes have been shown to promote the malignant transformation of recipient cells, but the mechanisms remain unclear.

**Methods:**

The level of miR-224-5p in CRC cell-derived exosomes was determined by RT-qPCR assay. In addition, PKH26 dye-labeled exosomes were used to assess the efficacy of the transfer of exosomes between SW620 and normal colon epithelial cell line CCD 841 CoN.

**Results:**

In this study, we found that overexpression of miR-224-5p significantly promoted the proliferation, migration, and invasion and inhibited the oxidative stress of SW620 cells. In addition, miR-224-5p can be transferred from SW620 cells to CCD 841 CoN cells via exosomes. SW620 cell-derived exosomal miR-224-5p markedly promoted proliferation, migration, and invasion of CCD 841 CoN cells. Meanwhile, SW620 cell-derived exosomal miR-224-5p notably decreased the expression of CMTM4 in CCD 841 CoN cells. Furthermore, SW620 cell-derived exosomal miR-224-5p significantly promoted tumor growth in a xenograft model *in vivo*.

**Conclusion:**

These findings suggested that SW620 cell-derived exosomal miR-224-5p could promote malignant transformation and tumorigenesis *in vitro* and *in vivo* via downregulation of CMTM4, suggesting that miR-224-5p might be a potential target for therapies in CRC.

## 1. Introduction

Colorectal cancer (CRC) is one of the most common digestive tract malignancies with high morbidity and mortality [1, 2]. The development and progression of CRC are regulated by several factors, such as dietary behaviors, chronic intestinal inflammation, aging, smoking, and mutations [3, 4]. It is estimated that around 1 million people will be affected by CRC every year, accompanied by overt metastatic disease [2]. Recently, several methods including radiotherapy and chemoradiotherapy have been used for the treatment of CRC for years [5–7]. However, the prognosis of patients with CRC is still unsatisfactory [5–7]. Therefore, identifying specific biomarkers is of great importance to improve early diagnosis and investigate novel treatment strategies for patients of CRC.

MicroRNAs (miRNAs) play an important role in the development of human cancers [8]. For example, MRX43 (miR-34a mimic) is the first synthetic tumor-targeted miRNA to enter a clinical trial [9, 10], suggesting that miRNAs are increasingly recognized as important regulators for the treatment of human cancers. Accumulating evidence has shown that miRNAs exert a vital role in regulating CRC progression through acting as tumor suppressors and oncogenes [8, 11]. Exosomes are small (30–100 nm) extracellular vesicles that carry different nucleic acids including miRNAs [12, 13]. In addition, exosomes serve as key mediators in cell-to-cell communication, often to prepare a premetastatic niche, remodel the extracellular environment, and escape immune surveillance [13, 14]. Recently, cancer cell-derived exosomes have been reported to influence tumor microenvironment and promote cancer cell growth, invasion, and angiogenesis [15–17]. Meanwhile, cancer cell-derived miRNAs can be delivered to recipient cells via exosomes, then perform a key regulatory role in migration and invasion of CRC cells [18, 19].

In this study, we found that miR-224-5p plays an important role in CRC. Overexpression of miR-224-5p could promote CRC cell proliferation, migration, and invasion via targeting CMTM4. CMTM4 is considered as a tumor suppressor in multiple cancers including CRC [20, 21]. Xue et al. found that overexpression of CMTM4 was able to suppress the proliferation and migration of CRC cells [20]. Furthermore, miR-224-5p can be transferred from CRC cells to CCD 841 CoN cells via exosomes. Meanwhile, exosomal miR-224-5p decreased the expression of CMTM4 in CCD 841 CoN cells, which resulted in the malignant transformation of colon epithelial cells. These findings may provide a theoretical basis for the research of CRC.

## 2. Materials and Methods

### 2.1. Data Collection and Differential Expression Analysis

CRC-related datasets (GSE18392, GSE115513, and GSE126093) were downloaded from the GEO database. The differentially expressed miRNAs (DEMs) between CRC tissues and adjacent normal tissues were identified using R language. The miRNAs with *P* < 0.05 and |log2 (FC)| > 2 were selected as the significantly DEMs. The overlapping DEMs were identified using a Venn diagram from three datasets (GSE18392, GSE115513, and GSE126093). In addition, TCGA dataset was used to determine the association between the overall survival of patients with CRC and miR-224-5p level.

### 2.2. Patient Samples

A total of 20 serum samples were collected from 10 patients with CRC and 10 healthy participants from the Harbin Medical University Cancer Hospital. Written informed consent was obtained from all participators. This study was approved by the ethics committee of the Harbin Medical University Cancer Hospital.

### 2.3. Cell Culture

Human normal colon epithelial cell line (CCD 841 CoN), CRC cell lines (HT-29, HCT116, SW620, and SW480), and 293T cells were obtained from the Type Culture Collection of the Chinese Academy of Sciences. HT-29 and HCT116 cells were maintained in McCoy's 5A medium (Thermo Fisher Scientific, Waltham, MA, USA) with 10% FBS (Thermo Fisher Scientific). SW620 and SW480 cells were maintained in Leibovitz's L-15 medium containing 10% FBS and cultured in a humidified incubator containing 5% CO_2_ at 37°C.

### 2.4. Cell Transfection

miRNA negative control (NC), miR-224-5p agomir, and miR-224-5p antagomir were obtained from RiboBio. Next, NC, miR-224-5p agomir, or miR-224-5p antagomir plasmids were transfected into SW620 and HCT116 cells, respectively, by using Lipofectamine 2000.

In addition, the sequence of CMTM4 was amplified by PCR and then subcloned into pcDNA3.1 vector to generate pcDNA3.1-CMTM4 overexpression (CMTM4-OE) plasmids. The pcDNA3.1-NC and CMTM4-OE plasmids were transfected into CCD 841 CoN cells by using Lipofectamine 2000 reagents. Subsequently, 48 h after transfection, the transfected cells were selected with neomycin (Thermo Fisher Scientific).

### 2.5. RT-qPCR Assay

The TRIpure Total RNA Extraction Reagent (ELK Biotechnology, Hubei, China) was used to extract total RNA from cells. After that, RNA (1 *μ*g) was reversely transcribed into cDNA using EntiLink™ 1st Strand cDNA Synthesis Kit (ELK). Later on, real-time PCR was carried out on a StepOne™ Real-Time PCR System using the EnTurbo™ SYBR Green PCR SuperMix kit (ELK). The primer sequences were as follows: *β*-actin, forward: 5′-GTCCACCGCAAATGCTTCTA-3′, reverse: 5′-TGCTGTCACCTTCACCGTTC-3′; CMTM4, forward: 5′-CTGCCGTGATATTTGGCTTCT-3′, reverse: 5′-CGGATGTAGTCATTGGTGCTCT-3′; U6, forward: 5′-CTCGCTTCGGCAGCACAT-3′, reverse: 5′-AACGCTTCACGAATTTGCGT-3′; miR-224-5p, forward: 5′-CAAGTCACTAGTGGTTCCGTTTAG-3′, reverse: 5′-CTCAACTGGTGTCGTGGAGTC-3′. The expression of CMTM4 was normalized to *β*-actin. The expression of miR-224-5p was normalized to U6.

### 2.6. Cell Viability Assay

The transfected cells were plated onto 96-well plates (about 5,000 per well). At 0, 24, 48, or 72 h posttransfection, CCK-8 reagent (10 *μ*l, MedChemExpress) was added into each well, and cells were then incubated for 2 h. After that, the absorbance of each well was detected using a microplate reader at 450 nm.

### 2.7. Immunofluorescence Assay

Cells were treated with 4% paraformaldehyde for 20 min and incubated with 5% BSA for 1 h. Then, cells were incubated with primary antibodies anti-Ki67 at 4°C overnight. After that, cells were incubated with a corresponding secondary antibody for 1 h. Subsequently, images were captured using a fluorescence microscope. Nuclei were stained by DAPI.

### 2.8. Transwell Assay

Transwell migration or invasion assays were performed using transwell chambers uncoated or coated with Matrigel. Cells (2 × 10^4^ cells/well) were seed onto the upper chamber of each insert (Corning). In addition, the lower chambers were loaded with DMEM (600 *μ*l) containing 10% FBS. After 24 h of incubation, the cells on the lower surface were stained with 0.1% crystal violet. Subsequently, the migrated or invasive cells were photographed in five random microscopic regions using a light microscope (magnification 200x).

### 2.9. Flow Cytometry Assay

Cell apoptosis was detected using an Annexin-V-FITC apoptosis detection kit (SUNGENE BIOTECH). Briefly, 1 × 10^5^ cells were stained with Annexin-V-FITC (5 *μ*l) and PI (5 *μ*l) staining solution for 15 min in darkness. Subsequently, cell apoptosis was analyzed by a flow cytometer.

### 2.10. ELISA Assay

The levels of superoxide dismutase (SOD) and glutathione (GSH) in SW620 cells were detected using ELISA kits (Nanjing Jiancheng Bioengineering Institute, Nanjing China).

### 2.11. Dual-Luciferase Reporter Assay

The 293T cells were cotransfected with pGL6-miR-based luciferase reporter plasmids containing wild-type (WT) or mutant (MT) 3′-UTR of CMTM4 and miR-224-5p agomir using Lipofectamine 2000. Later on, the luciferase activity in cell lysates was measured by the Dual-Luciferase Reporter Assay System (Beyotime, Beijing, China).

### 2.12. Exosome Isolation and Characterization

The conditioned media (CM) of SW620 cells was collected. After that, exosomes were isolated using the GETTM Exosome Isolation Kit (GeneExosome technologies). Nanoparticle-tracking analysis (Particle Metrix, Meerbusch, Germany) was applied to determine the size of exosomes. Next, a transmission electron microscopy (TEM) was used to visualize the morphology of exosomes as described previously [22].

### 2.13. Exosome Labeling and Uptake

SW620 cell-derived exosomes were mixed with PKH26 dye for 30 min. After that, PKH26-tagged exosomes were added into CCD 841 CoN cells and incubated for 24 h. Subsequently, CCD 841 CoN cells that uptake the labelled exosomes were observed by a fluorescence microscope. Nuclei were stained by DAPI.

### 2.14. Western Blot Assay

Proteins were separated by 10% SDS-PAGE and then transferred onto a PVDF membrane. Later on, the membrane was incubated overnight at 4°C with primary antibodies against CMTM4, p-Akt, Akt, p-ERK, ERK, CD63, TSG101, and *β*-actin and then incubated with the corresponding secondary antibody at room temperature for 1 h. Subsequently, immune complexes were detected using the ECL reagents.

### 2.15. Animal Studies

The BALB/c nude mice (4–5 weeks old) were purchased from the Vital River Laboratories (Beijing, China). SW620 cells (1 × 10^7^ cells) were subcutaneously injected into left flank of nude mice. When the tumors reach about 200 mm^3^, mice were divided randomly into four groups: control, Exo-NC, Exo-miR-224-5p agomir, and Exo-miR-224-5p antagomir. Then, mice were intravenously injected with PBS, Exo-NC, Exo-miR-224-5p agomir, or Exo-miR-224-5p antagomir twice a week. The tumor size was measured with a vernier caliper every week, and the volume was calculated by the following formula *V* = length × width^2^ × 0.5. After 3 weeks of tumor cell implantation, the mice were sacrificed via an overdose of CO_2_ (30% volume/min), and the tumors from different groups were removed and weighted. All animal experiments were approved by the Ethics Committee of the Harbin Medical University Cancer Hospital and performed following the procedures of National Institutes of Health guide for the care and use of laboratory animals.

### 2.16. TUNEL Assay

Cell apoptosis in tumor tissues was assessed using an APO-BrdU™ TUNEL Assay Kit (Thermo Fisher Scientific) according to the manufacturer's instructions.

### 2.17. Statistical Analysis

All statistical analyses were performed using the GraphPad Prism software (version 7.0, La Jolla, CA, USA). Differences between three or more groups were analyzed by one-way analysis of variance (ANOVA) and Tukey's tests. Data are presented as mean ± standard deviation (S.D.). The differences were considered significant at ∗*P* < 0.05. All data were repeated in triplicate.

## 3. Results

### 3.1. Identification of DEMs in CRC

**Figure 1:**
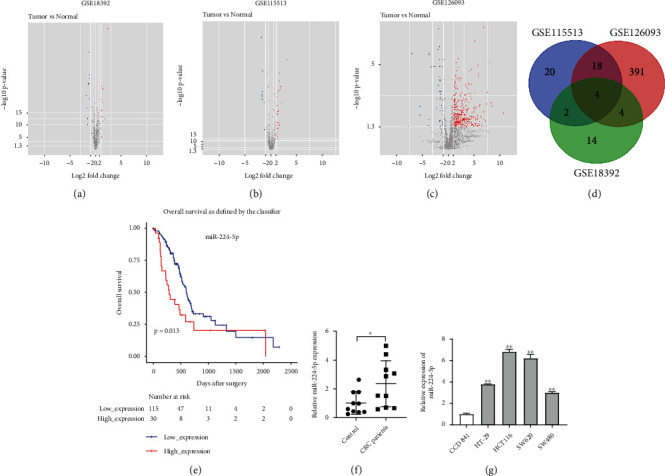
Identification of DEMs in CRC. (a–c) The volcano plot shows the DEMs between CRC tissues and adjacent normal tissues in the GSE18392, GSE115513, and GSE126093 datasets. (d) Venn diagram of overlapping DEMs from intersection of GSE18392, GSE115513, and GSE126093 datasets. (e) The correlation between the level of miR-224-5p and overall survival rate in patients with CRC in the TCGA dataset. (f) RT-qPCR assay was used to determine the level of miR-224-5p in serum samples from patients with CRC and healthy controls. ^∗^*P* < 0.05. (g) miR-224-5p levels in CRC cells (HT-29, HCT116, SW620, and SW480) and CCD 841 CoN cells were detected by RT-qPCR. ^∗∗^*P* < 0.01 vs. CCD 841 CoN group.

To identify the DEMs between CRC tissues and adjacent normal tissues, R language was performed to analyze the expression profiles of miRNAs from three CRC-related datasets (GSE18392, GSE115513, and GSE126093). As shown in Figures 1(a)–1(c), a total of 24, 44, and 417 DEMs were identified from the GSE18392, GSE115513, and GSE126093, respectively. In addition, 4 overlapping DEMs were identified in three datasets, including miR-31-5p, miR-135-5p, miR-183-5p, and miR-224-5p (Figure 1(d)). Zheng et al. revealed that miR-224 level was increased in CRC tissues [23]. Moreover, in the TCGA dataset, high level of miR-224-5p in patients with CRC is associated with a shorter overall survival, indicating that increased miR-224-5p level may predict poor overall survival in patients with CRC (Figure 1(e)). Meanwhile, the level of miR-224-5p in serum samples was higher in patients with CRC compared with that in healthy controls (Figure 1(f)). Furthermore, miR-224-5p level was markedly increased in HT-29, HCT116, SW620, and SW480 cells compared with CCD 841 CoN cells (Figure 1(g)). HCT116 and SW620 cell lines exhibited higher level of miR-224-5p (Figure 1(g)). To sum up, the level of miR-224-5p is upregulated in CRC and is associated with a poor prognosis.

### 3.2. Overexpression of miR-224-5p Promoted the Proliferation, Migration, and Invasion of CRC Cells

**Figure 2:**
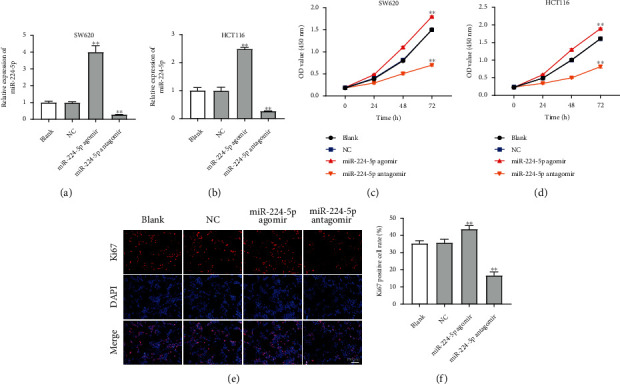
Overexpression of miR-224-5p increased the viability and proliferation of CRC cells. (a, b) SW620 and HCT116 cells were transfected with NC, miR-224-5p agomir, or miR-224-5p antagomir. RT-qPCR was applied to detect the level of miR-224-5p in SW620 and HCT116 cells. (c, d) The viability of SW620 and HCT116 cells was determined by CCK-8 assay. (e, f) Cell proliferation detected in SW620 cells using Ki67 immunofluorescence assay. ^∗∗^*P* < 0.01 vs. NC group.

**Figure 3:**
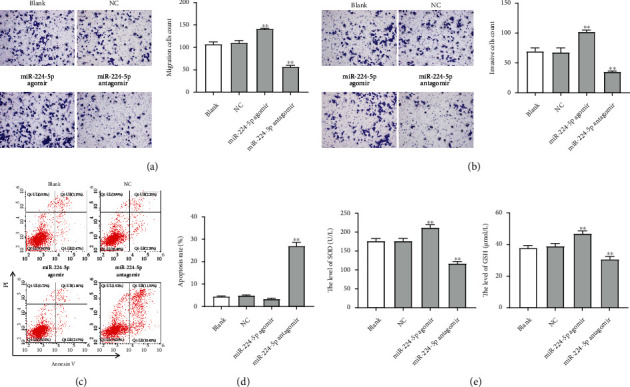
Overexpression of miR-224-5p promoted the migration and invasion of CRC cells. SW620 cells were transfected with NC, miR-224-5p agomir, or miR-224-5p antagomir. (a) Transwell migration assay was applied to assess cell migration. (b) Transwell invasion assay was conducted to determine cell migration. (c, d) Cell apoptosis was determined using flow cytometry assay with Annexin V and PI double staining. (e) ELISA assay was used to determine the levels of SOD and GSH in SW620 cells. ^∗∗^*P* < 0.01 vs. NC group.

To investigate the role of miR-224-5p in CRC cells, SW620 and HCT116 cells were transfected with miR-224-5p agomir or antagomir. As shown in Figures 2(a) and 2(b), miR-224-5p agomir notably increased miR-224-5p level in SW620 and HCT116 cells, while miR-224-5p antagomir displayed the opposite results. Moreover, miR-224-5p agomir notably promoted the viability and proliferation of SW620 and HCT116 cells, while miR-224-5p antagomir obviously suppressed cell viability and proliferation (Figures 2(c)–2(f)). Meanwhile, as shown in Figures 3(a) and 3(b), miR-224-5p agomir significantly promoted the migration and invasion of SW620 cells. In contrast, miR-224-5p antagomir suppressed SW620 cell migration and invasion and triggered cell apoptosis (Figures 3(a)–3(d)). Furthermore, miR-224-5p agomir markedly increased the levels of GSH and SOD in SW620 cells, while miR-224-5p antagomir exhibited the opposite effects (Figure 3(e)). Collectively, overexpression of miR-224-5p could promote the proliferation, migration, and invasion and inhibit the oxidative stress of CRC cells.

### 3.3. CMTM4 Is a Binding Target of miR-224-5p

**Figure 4:**
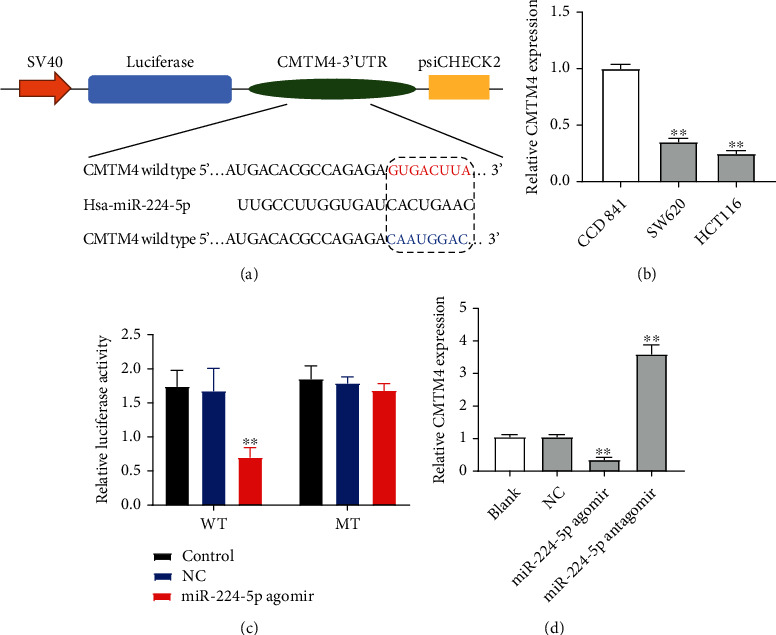
CMTM4 is a direct binding target of miR-224-5p. (a) Schematic diagram of binding sites between miR-224-5p and CMTM4 and the mutation of binding sites in CMTM4. (b) The levels of CMTM4 in CCD 841 CoN, SW620, and HCT116 cells were assessed by RT-qPCR. ^∗∗^*P* < 0.01 vs. CCD 841 CoN group. (c) Luciferase reporter assay in SW620 cells cotransfected with WT or MT CMTM4 3′UTR reporter gene and NC or miR-224-5p. (d) RT-qPCR analysis of CMTM4 level in SW620 cells transfected with NC, miR-224-5p agomir, or miR-224-5p antagomir. ^∗∗^*P* < 0.01 vs. NC group.

Evidence has shown that miRNAs can negatively regulate protein expression though binding to the 3′-UTR of their target mRNA [24, 25]. To find the target genes of miR-224-5p, five bioinformatics tools (miRWalk, miRanda, miRDB, RNA22, and TargetScan) were used. We found that CMTM4 was one of the best candidates (Figure 4(a)). It has been shown that CMTM4 level was frequently downregulated in multiple cancers including CRC [20, 21]. In addition, overexpression of CMTM4 was able to suppress the proliferation and migration of CRC cells [20]. As predicted, a marked decrease in CMTM4 at mRNA level was found in SW620 and HCT116 cells (Figure 4(b)). To validate that CMTM4 is a binding target of miR-224-5p, dual-luciferase reporter assay was used. As shown in Figure 4(c), miR-224-5p agomir notably reduced the luciferase activity of CMTM4-WT. Meanwhile, miR-224-5p agomir obviously decreased the expression of CMTM4 at mRNA level in SW620 cells, while miR-224-5p antagomir displayed the opposite results (Figure 4(d)). These results indicated that CMTM4 is a direct binding target of miR-224-5p.

### 3.4. MiR-224-5p Can Be Transferred from SW620 Cells to CCD 841 CoN Cells via Exosomes

**Figure 5:**
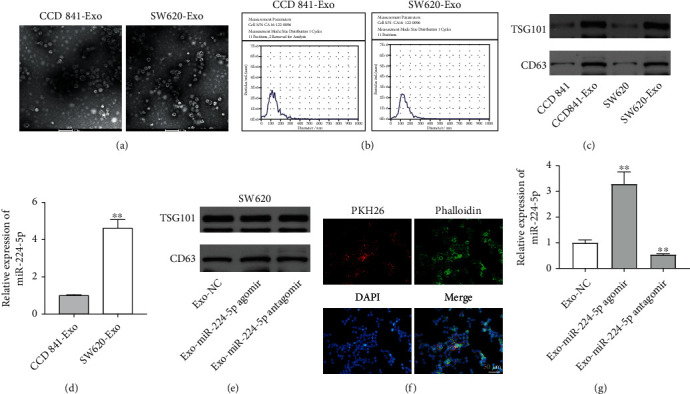
miR-224-5p can be transferred from SW620 cells to CCD 841 CoN cells via exosomes. (a) TEM of CCD 841 CoN cell and SW620 cell CM secreted exosomes. (b) NTA was used to determine exosome number and size distribution. (c) Western blot analysis of exosomal proteins TSG101 and CD63 in CCD 841 CoN cells, CCD 841 CoN cell-derived exosomes, and SW620 cells, SW620 cell-derived exosomes. (d) RT-qPCR analysis of miR-224-5p level in CCD 841 CoN cell-derived exosomes and SW620 cell-derived exosomes. (e) SW620 were transfected with NC, miR-224-5p agomir, or miR-224-5p antagomir for 48 h. Western blot analysis of TSG101 and CD63 levels in exosomes isolated from the transfected SW620 cells. (f) CCD 841 CoN cells were cocultured with SW620 cell-derived exosomes for 48 h. The uptake of exosomes (red color) into CCD 841 CoN cells was observed by confocal microscopy. (g) RT-qPCR analysis of miR-224-5p level in exosomes isolated from the transfected SW620 cells. ^∗∗^*P* < 0.01 vs. the CCD 841 CoN group or the Exo-NC group.

Evidence has shown that cancer cell-derived exosomes exert a vital role in the process of malignant transformation [26, 27]. In this study, exosomes were isolated from the CM of CCD 841 CoN cells and SW620 cells. TEM and NTA analysis demonstrated that exosomes secreted from CCD 841 CoN and SW620 cells had the characteristic size (40 to 100 nm) and cup-shaped morphology and expressed the exosomal markers, TSG101 and CD63 (Figures 5(a)–5(c)), suggesting that exosomes were isolated from cells. Meanwhile, miR-224-5p level was increased in exosomes derived from SW620 cells (SW620-Exo) compared with that in exosomes derived from CCD 841 CoN cells (CCD 841-Exo) (Figure 5(d)).

We further explored whether miR-224-5p can be transferred from SW620 cells to CCD 841 CoN cells via exosomes. First, exosomes were isolated from SW620 cells that were transfected with NC, miR-224-5p agomir, or miR-224-5p antagomir (Exo-NC, Exo-miR-224-5p agomir, and Exo-miR-224-5p antagomir). As revealed in Figure 5(e), these exosomes expressed the exosomal markers, TSG101 and CD63. Next, to determine whether CCD 841 CoN cells could take up SW620 cell-derived exosomes, CCD 841 CoN cells were cocultured with PKH26-labeled exosomes. As revealed in Figure 5(f), PKH26 fluorescence dye was observed in CCD 841 CoN cells. In addition, miR-224-5p level was upregulated in CCD 841 CoN cells incubated with Exo-miR-224-5p agomir, whereas CCD 841 CoN cells cocultured with Exo-miR-224-5p antagomir displayed the opposite results (Figure 5(g)). Collectively, oligonucleotide sequences (miR-224-5p agomir or miR-224-5p antagomir) could be transferred from SW620 cells to CCD 841 CoN cells via exosomes.

### 3.5. Intercellular Transfer of miR-224-5p Agomir by Exosomes Promoted Malignant Transformation of CCD 841 CoN Cells via Downregulation of CMTM4

**Figure 6:**
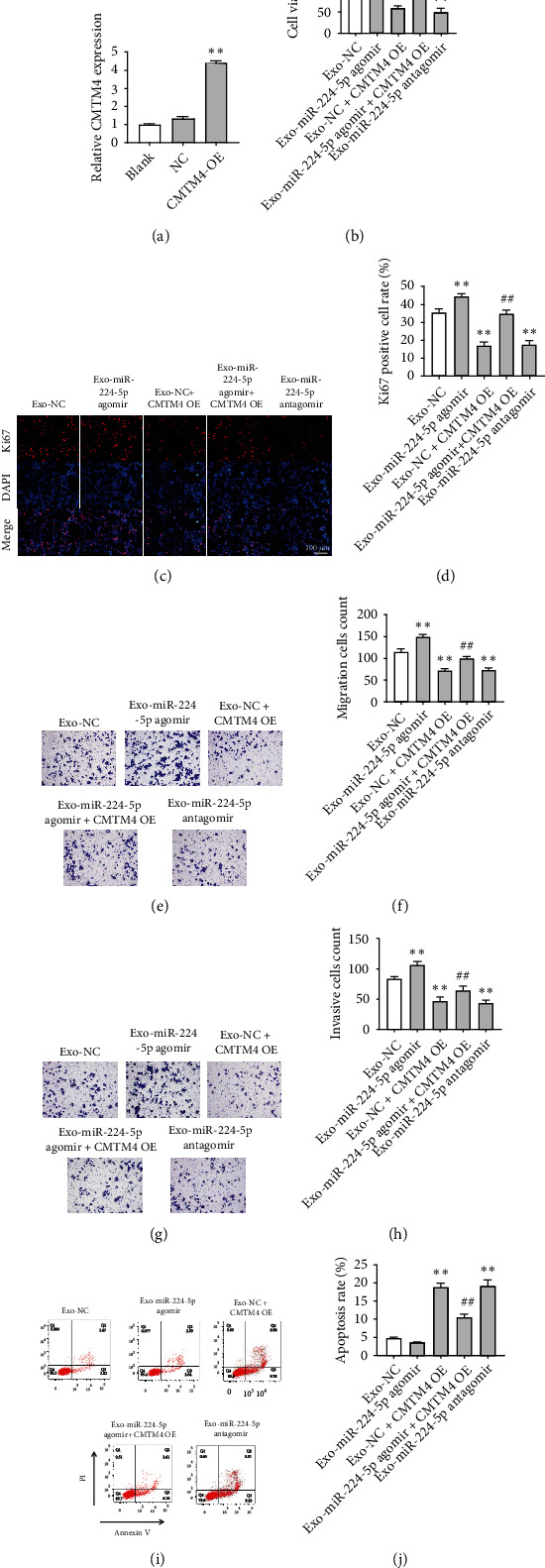
Intercellular transfer of miR-224-5p agomir by exosomes promoted malignant transformation of CCD 841 CoN cells via downregulation of CMTM4. (a) RT-qPCR analysis of CMTM4 expression in CCD 841 CoN cells transfected with CMTM4-OE. ^∗∗^*P* < 0.01 vs. NC group. (b) SW620 cells were transfected with NC, miR-224-5p agomir, or miR-224-5p antagomir for 48 h. CCD 841 CoN cells were cocultured with exosomes isolated from the transfected SW620 cells in the presence or absence of CMTM4-OE. Cell viability was determined by CCK-8 assay. (c, d) Cell proliferation detected using Ki67 immunofluorescence assay. (e, f) Transwell migration assay was applied to assess cell migration. (g, h) Transwell invasion assay was conducted to determine cell migration. (i, j) Cell apoptosis was determined using flow cytometry assay with Annexin V and PI double staining. ^∗∗^*P* < 0.01 vs. the Exo-NC group; ^##^*P* < 0.01 vs. the Exo-miR-224-5p agomir group.

**Figure 7:**
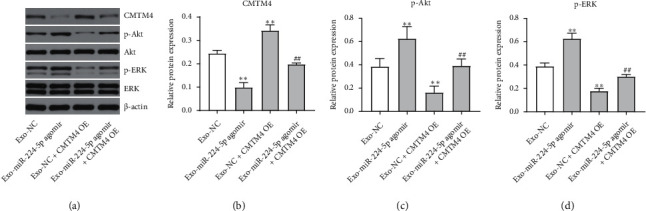
Intercellular transfer of miR-224-5p agomir by exosomes promoted malignant transformation of CCD 841 CoN cells via CMTM4/Akt/ERK pathway. SW620 cells were transfected with NC, miR-224-5p agomir, or miR-224-5p antagomir for 48 h. CCD 841 CoN cells were cocultured with exosomes isolated from the transfected SW620 cells in the presence or absence of CMTM4-OE. (a–d) CMTM4, p-Akt, and p-ERK expressions in CCD 841 CoN cells were detected with western blotting assay. ^∗∗^*P* < 0.01 vs. the Exo-NC group; ^##^*P* < 0.01 vs. the Exo-miR-224-5p agomir group.

Next, we investigated the role of Exo-miR-224-5p agomir or Exo-miR-224-5p antagomir in recipient cells. As revealed in Figure 6(a), CMTM4 level was markedly upregulated in CCD 841 CoN cells after transfection with CMTM4-OE. In addition, Exo-miR-224-5p agomir significantly promoted the viability, proliferation, migration, and invasion of CCD 841 CoN cells compared with the Exo-NC group; however, these Exo-miR-224-5p agomir-induced changes were inhibited when CCD 841 CoN cells were transfected with CMTM4-OE plasmids (Figures 6(b)–6(h)). In contrast, Exo-miR-224-5p antagomir notably suppressed the viability, proliferation, migration, and invasion and triggered the apoptosis of CCD 841 CoN cells compared with the Exo-NC group (Figures 6(b)–6(j)). Moreover, Exo-miR-224-5p agomir markedly downregulated the expression of CMTM4 and upregulated p-Akt and p-ERK protein expressions in CCD 841 CoN cells; however, these phenomena were reversed when CCD 841 CoN cells were transfected with CMTM4-OE plasmids (Figures 7(a)–7(d)). These data suggested that exosomal miR-224-5p is involved in the malignant transformation of CCD 841 CoN cells.

### 3.6. Exo-miR-224-5p Antagomir Suppressed the Growth of CRC Cells *In Vivo*

**Figure 8:**
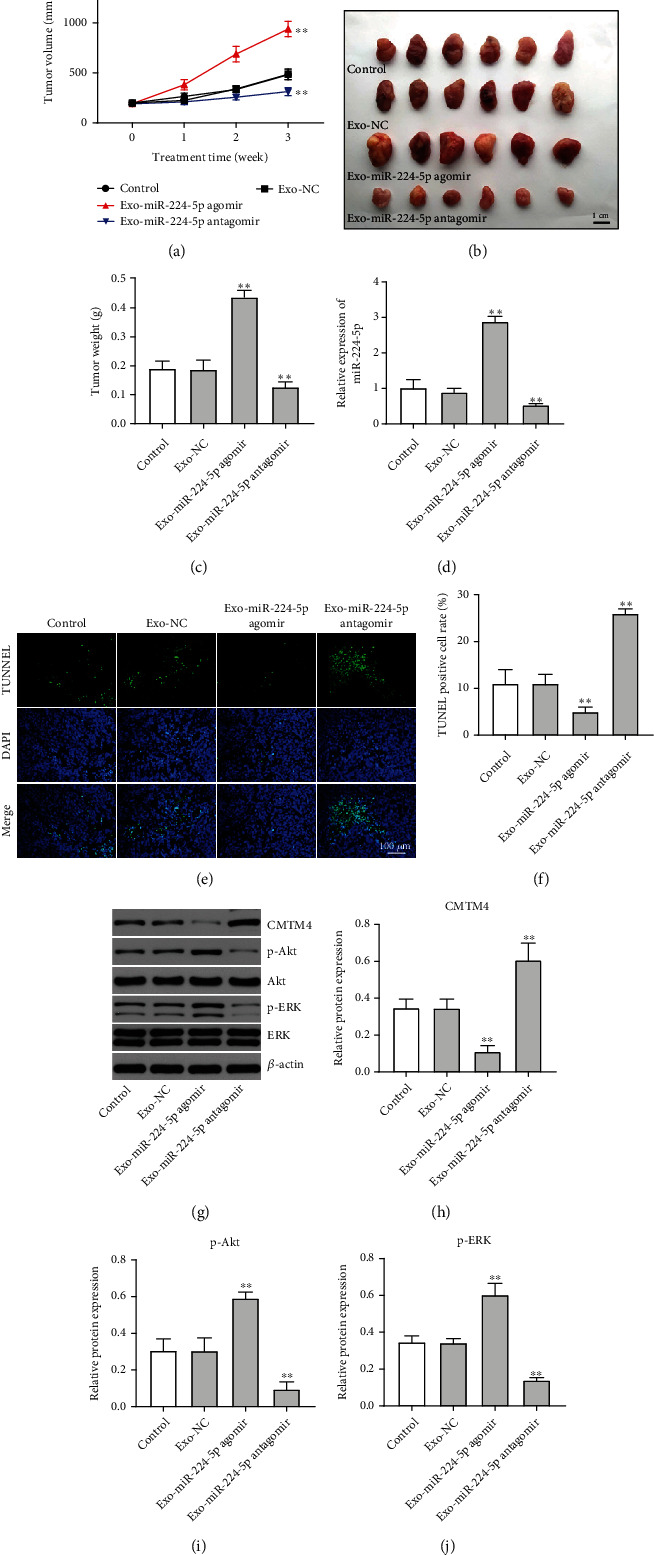
Exo-miR-224-5p antagomir suppressed the growth of CRC cells *in vivo.* (a) The tumor volume was measured every week. (b, c) Tumors were removed and weighted. (d) RT-qPCR was used to determine the level of miR-224-5p in tumor tissues. (e, f) Cell apoptosis in tumor tissues was analyzed using TUNEL assay. (g–j) CMTM4, p-Akt, and p-ERK expressions in tumor tissues were detected with western blotting assay. ^∗∗^*P* < 0.01 vs. the Exo-NC group.

We further investigated the role of exosomal miR-224-5p on tumor growth *in vivo*. As shown in Figures 8(a)–8(c), the tumor volume and tumor weight were significantly increased in the Exo-miR-224-5p agomir group, while the opposite results were observed in the Exo-miR-224-5p antagomir group. In addition, the level of miR-224-5p was upregulated in tumor tissues of tumor-bearing mice that received Exo-miR-224-5p agomir, whereas mice that received Exo-miR-224-5p antagomir displayed the opposite results (Figure 8(d)). Moreover, TUNEL assay indicated that Exo-miR-224-5p antagomir obviously induced cell apoptosis in tumor tissues (Figures 8(e) and 8(f)). Meanwhile, the expression of CMTM4 was decreased, and p-Akt and p-ERK protein expressions were increased in tumor tissues of mice that received Exo-miR-224-5p agomir compared with the Exo-NC group, whereas mice that treated with Exo-miR-224-5p antagomir displayed the opposite results (Figures 8(g)–8(j)). Collectively, Exo-miR-224-5p antagomir could suppress the growth of CRC cells *in vivo* via upregulation of CMTM4.

## 4. Discussion

The development of human cancers is a complex process [28]. Tumor cells are the major driving force behind the development and progression of human cancers [29]. García-Olmo found that interactions between tumor cells and neighboring normal cells in the tumor microenvironment might be essential for tumor progression [30]. Local microenvironments have been found to exert a vital role in mediating intercellular communication between malignant cells and nonmalignant cells [31, 32]. In addition, cancer cell-derived exosomes can act as communicative vectors, participating in remodeling the tumor microenvironment [33, 34]. Importantly, cancer cell-derived exosomes have been shown to promote the malignant transformation of recipient cells, increasing cell migratory and invasive abilities [35]. Meanwhile, cancer cell-derived exosomes can carry different miRNAs, and these exosomal miRNAs can modulate the function of recipient cells via activation or inactivation of multiple cancer related pathways through transferring into recipient cells and mediating protein expression [36, 37]. Thus, the discovery of novel circulating biomarkers of CRC may help to improve diagnosis or treatment of CRC.

Currently, miR-224-5p has been identified to be dysregulated in human cancers, including CRC [23, 38]. In this study, we found that miR-224-5p level was significantly upregulated in CRC cells, and downregulation of miR-224-5p could induce cell apoptosis and inhibit cell migration and invasion. Consistent with our present results, Zheng et al. showed that miR-224 overexpression promoted CRC cell proliferation and migration via targeting BTRC [23]. In addition, Liang et al. showed that downregulation of miR-224 could suppress the proliferation and trigger the apoptosis of Adriamycin-resistant CRC cells [39]. Our data found that miR-224-5p may act as an oncogene for CRC.

For the first time, we found that exosomal miR-224-5p is secreted by SW620 cells that could be internalized by CCD 841 CoN cells, suggesting that SW620-secreted miR-224-5p can be delivered into CCD 841 CoN cells via exosomes. A series of functional experiments indicated that exosomal miR-224-5p derived from CRC cells could promote CCD 841 CoN cell proliferation, migration, and invasion. Consistent with our present results, Wei et al. showed that miR-15b-3p can be transferred from GC cells to normal GES-1 gastric epithelium cells via exosomes and then promoted GES-1 cell malignant transformation [40]. In addition, evidence has shown that inactivation of tumor-suppressor and activation of oncogene are considered as the key causes driving the progressive transformation of normal cells to malignant cells [29]. In the present study, we found that CMTM4 was a binding target of miR-224-5p. It has been shown that CMTM4 is a tumor suppressor in human cancers, including CRC [20, 21]. Our data showed that exosomal miR-224-5p promoted the proliferation, migration, and invasion of CCD 841 CoN cells via downregulation of CMTM4. These results suggested that SW620 cell-derived exosomal miR-224-5p could promote the malignant transformation of CCD 841 CoN cells via inactivation of a tumor suppressor CMTM4.

The present study had some limitations. First, it has been shown that exosomal miRNAs play an important role in regulating the chemosensitivity of cancer cells [19, 41]. Thus, it is important for us to investigate the association between exosomal miR-224-5p and chemosensitivity of CRC cells in the future. Second, although we found that the level of miR-224-5p is upregulated in CRC cells and is associated with a poor prognosis, a question that remains to be answered is whether miR-224-5p level is associated with both tissue microenvironment and age of patients with CRC. Third, miR-224-5p can regulate numerous genes at the same time; thus, further studies are needed to investigate whether miR-224-5p can regulate CRC progression via targeting other gene (e.g., HIPK2) [42]. Finally, there are some challenges in the clinical translation of exosomal miRNAs, such as massive production, drug loading, and quality control [43].

## 5. Conclusion

Collectively, our data showed that exosomes secreted from SW620 cells can deliver miR-224-5p into CCD 841 CoN cells, promoting malignant transformation of CCD 841 CoN cells via downregulation of CMTM4. These findings may provide a theoretical basis for the research of CRC.

## Data Availability

The datasets used and/or analyzed during the current study are available from the corresponding author on reasonable request.
